# C-terminal residues of skeletal muscle calsequestrin are essential for calcium binding and for skeletal ryanodine receptor inhibition

**DOI:** 10.1186/s13395-015-0029-7

**Published:** 2015-02-22

**Authors:** Nicole A Beard, Angela F Dulhunty

**Affiliations:** John Curtin School of Medical Research, Australian National University, Garran Road, Canberra, ACT 2601 Australia; Discipline of Biomedical Sciences, Centre for Research in Therapeutic Solutions, Faculty of Education Science, Technology and Maths, University of Canberra, Kirinari Street, Bruce, ACT 2601 Australia

**Keywords:** Calsequestrin, Sarcoplasmic reticulum, Ryanodine receptor, Ca^2+^ binding protein, Skeletal muscle

## Abstract

**Background:**

Skeletal muscle function depends on calcium signaling proteins in the sarcoplasmic reticulum (SR), including the calcium-binding protein calsequestrin (CSQ), the ryanodine receptor (RyR) calcium release channel, and skeletal triadin 95 kDa (trisk95) and junctin, proteins that bind to calsequestrin type 1 (CSQ1) and ryanodine receptor type 1 (RyR1). CSQ1 inhibits RyR1 and communicates store calcium load to RyR1 channels via trisk95 and/or junctin.

**Methods:**

In this manuscript, we test predictions that CSQ1’s acidic C-terminus contains binding sites for trisk95 and junctin, the major calcium binding domain, and that it determines CSQ1’s ability to regulate RyR1 activity.

**Results:**

Progressive alanine substitution of C-terminal acidic residues of CSQ1 caused a parallel reduction in the calcium binding capacity but did not significantly alter CSQ1’s association with trisk95/junctin or influence its inhibition of RyR1 activity. Deletion of the final seven residues in the C-terminus significantly hampered calcium binding, significantly reduced CSQ’s association with trisk95/junctin and decreased its inhibition of RyR1. Deletion of the full C-terminus further reduced calcium binding to CSQ1 altered its association with trisk95 and junctin and abolished its inhibition of RyR1.

**Conclusions:**

The correlation between the number of residues mutated/deleted and binding of calcium, trisk95, and junctin suggests that binding of each depends on diffuse ionic interactions with several C-terminal residues and that these interactions may be required for CSQ1 to maintain normal muscle function.

## Background

In skeletal muscle, the rapid and co-ordinated release of calcium ions from the internal sarcoplasmic reticulum (SR) store is essential in triggering muscle contraction*.* Skeletal muscle activation, in response to an action potential on the surface membrane, activates voltage-gated L-type Ca^2+^ channels which, in turn, initiate SR Ca^2+^ release. The SR Ca^2+^ release channel is the ligand-gated ryanodine receptor (RyR), a large (>2 mDa) homotetrameric ion channel, which releases between 10% to 17% of the total SR Ca^2+^ with each action potential (reviewed in [1]). Ryanodine receptor type 1 (RyR1) is central to excitation-contraction coupling, and its activity and ability to release Ca^2+^ is refined by the level of Ca^2+^ load inside the SR and by a luminal SR protein complex, including calsequestrin (CSQ), skeletal triadin 95 kDa (trisk95), and junctin.

CSQ is the most abundant Ca^2+^ buffering protein found within the SR, with its concentration reported to be between 11 and 36 μmol (l fiber volume)^−1^, dependent on muscle type [2]. Calsequestrin type 1 (CSQ1) is the only isoform expressed in fast twitch muscle fibers, while equal amounts of CSQ1 and the so-called cardiac CSQ2 isoform are expressed in slow twitch fibers [2]. Both isoforms display a high degree of homology, with the C-terminal tail extended in CSQ2. CSQ1 is a low affinity, moderate to high-capacity Ca^2+^-binding protein, binding between 50 and 80 mol Ca^2+^/mole CSQ1 with a *K*_D_ of approximately 1 to 2 mM [3,4]. CSQ1 buffers free SR Ca^2+^ to approximately 1 mM during the contraction/relaxation cycle.

Research over the past 2 decades has shown that CSQ1 plays additional roles in global regulation of Ca^2+^ signaling in skeletal and cardiac muscles. A retrograde signal from CSQ1 is thought to be important for store-operated Ca^2+^ entry in skeletal muscle [5]. CSQ1 inhibits RyR1, while in the heart, CSQ2 activates the cardiac RyR [6]. CSQ is considered to be a luminal Ca^2+^ sensor for the RyR, communicating Ca^2+^ store load to the channel [7-9]. In skeletal muscle, CSQ1 acts as a break on the channel, to curtail SR Ca^2+^ release under conditions of lower store load and is essential for normal muscle function. Knockout of CSQ1 leads to a malignant hyperthermia phenotype with excess Ca^2+^ release under stress [10]. Despite the functional interaction between CSQ1 and RyR1, there is no evidence that they bind directly *in vivo*. Instead, both proteins bind to junctin and trisk95 [11-16], forming a luminal protein complex, which allows CSQ1-RyR1 communication. The importance of the luminal protein complex in modulating RyR activity and excitation-contraction coupling is highlighted by the fact that mutation or alteration in expression of these proteins results in severe contractile dysfunction [17-23].

At *in vivo* luminal [Ca^2+^]s, CSQ1 monomers self-associate to form polymers, which are observed as long linear strand-like structures closely associated with the junctional face membrane in electron micrographs [24]. The dynamic polymerization of CSQ1 is highly dependent on Ca^2+^. At low [Ca^2+^] of 100 nM, CSQ1 exists in an unfolded randomly coiled structure [25]. As [Ca^2+^] is increased toward 100 μM, CSQ1 undergoes a conformational change [25-27] and increases in helicity [26,28]. The conformational changes include the folding of the three thioredoxin domains within CSQ1 and the subsequent formation of front-to-front dimers between two folded monomers [27]. In this process, the N-terminus from one monomer inserts into a groove between two β-strands of domain II of the second monomer [27]. A secondary intermolecular interaction among front-to-front dimers allows them to associate in a back-to-back configuration, bringing together two electronegative surfaces to form linear polymers [27].

Several factors influence CSQ1’s moderate to high Ca^2+^ binding capacity. Ca^2+^ binding capacity is partially determined by overall net charge [29]; however, the calculated net charge of skeletal CSQ1 is insufficient for the amount of Ca^2+^ that it can bind in its polymer form [30]. This suggests that additional sites for Ca^2+^ binding must be formed as CSQ1 polymerizes. The roles of the acidic-rich C-terminal tail (residues 354 to 367 in rabbit) has been postulated to play an important role in inferring the Ca^2+^ binding capacity of CSQ1 [15,29,31], although the mechanisms have not been well understood. Recent molecular dynamic modeling studies postulate that Ca^2+^ binding sites within the C-terminus of CSQ1 serve to neutralize the interface and enable CSQ polymerization and subsequent high-capacity Ca^2+^ binding [32]. Furthermore, the existence of low-affinity Ca^2+^ binding sites on CSQ1 may serve, at least in part, to induce additional Ca^2+^ binding sites [32], allowing for CSQ’s high-capacity Ca^2+^ binding. That the C-terminal tail is essential for binding capacity is supported experimentally, as its deletion in CSQ2 severely reduces Ca^2+^ binding capacity [29]. The C-terminal tail contains the highest surface negative charge density [30] and was initially thought to be responsible for at least 26% of the Ca^2+^ binding, although simulations suggest this to be as low as 10% [32].

CSQ1’s acidic-rich C-terminal tail also constitutes a “hot spot” for CSQ1 protein associations. CSQ1 residues 354 to 367 contain the binding site for trisk95 and have been suggested to contain critical residues that support its interaction with junctin [15]. We are yet to understand the true nature of this region, because the anatomical details of the acidic C-terminal tail are not resolved in published crystal structures of CSQ1 (reviewed in [33]) and because there is little information on the role of specific residues within the C-terminal tail in the various functions of CSQ1.

In this paper, we have dissected the functional importance of residues within CSQ1’s C-terminal tail. We have examined the effects of substitutions or deletions within the C-terminal tail on CSQ1’s Ca^2+^ binding capacity, its ability to associate with trisk95 and junctin and its ability to regulate RyR1 channel activity to ensure normal muscle function.

## Methods

### Materials

The monoclonal 34C anti-RyR1 antibody, monoclonal VIIID12 anti-CSQ1 antibody, and rabbit affinity-isolated polyclonal anti-CSQ antibody (ab 3516) were from Abcam (Cambridge, MA, USA). Polyclonal anti-junctin was a generous gift from Dr. Steven Cala (Wayne State University, MI, USA). Phospholipids were from Avanti Polar Lipids (Alabaster, AL, USA). The Pierce IP kit was from ThermoFisher Scientific (Scoresby, Vic, Australia), ^45^Ca^2+^ was from PerkinElmer (Glen Waverley, VIC, Australia), and Glutathione Sepharose 4B was from GE Healthcare (Rydalmere, NSW, Australia). The Bio-Rad DC protein determination assay and sodium dodecyl sulphate-polyacrylamide gel electrophoresis (SDS-PAGE)/Western blot apparatus and consumables were from Bio-Rad (Gladesville, NSW, Australia), and the multi-mutagenesis kit was from Stratagene (now Agilent Technologies Inc, Mulgrave, VIC, Australia). Microcon centrifugal filter concentrators were from Millipore (Bayswater, VIC, Australia). The monoclonal anti-triadin antibody (IIG12) and all other chemicals were obtained from Sigma-Aldrich (Castle Hill, NSW, Australia).

### Ethics approval

All animal work was approved by the Australian National University and University of Canberra Animal Ethics Committees.

### CSQ1 mutation, expression, and purification

**Figure 1:**
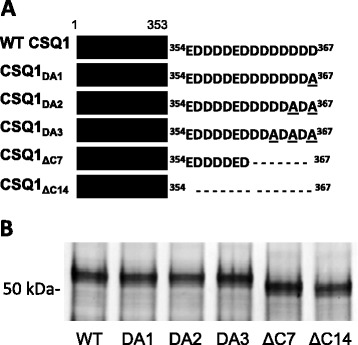
**CSQ1 mutation and expression. (A)** Diagram of the CSQ1 alanine point mutations and deletion mutants. **(B)** SDS-PAGE and silver stain of purified CSQ1 WT and mutant constructs. From left to right, WT CSQ1, CSQ1_DA1_, CSQ1_DA2_, CSQ1_DA3_, CSQ1_ΔC7_, and CSQ1_ΔC14_. Molecular weight markers are to the left of the blot in **(B)**.

To probe the functional importance of the acidic-rich C-terminal tail in CSQ1, wild-type (WT) CSQ1 and several alanine substitution and deletion mutants (see Figure 1) were generated by PCR of rabbit CSQ1 cDNA using the Stratagene multi-mutagenesis kit. All CSQ1 constructs were subcloned into a pGEX5x1 vector, containing a N-terminal glutathione S-transferase (GST) tag and expressed as previously described [34]. The expressed CSQ1 proteins were dialyzed against 20 mM MOPS, 150 mM NaCl, and 1 mM CaCl_2_ (pH 7.4). Where necessary, the protein underwent secondary purification using anti-CSQ1 immunoprecipitation (Pierce crosslinking IP kit), following manufacturer’s instruction.

### SR vesicle preparation

SR vesicles from back and leg muscles from New Zealand White Rabbits were prepared as described in [7,9,35].

RyR1 purification from skeletal SR vesicles was performed as described in [36]. The purified RyR1 was concentrated, snap-frozen, and stored at −70°C. The protein was run on SDS polyacrylamide gels, and immunoblots probed with anti-RyR1, anti-CSQ1, anti-triadin and anti-junctin antibodies to detect contamination by these proteins.

SDS-PAGE and Western blot were performed according to [37,38]. Briefly, proteins were separated on 4% to 15% or 4% to 20% SDS polyacrylamide gels and transferred to polyvinylidene difluoride (PVDF) membrane for Western blot. PVDF membranes were exposed to primary antibodies to CSQ1, junctin, trisk95, and RyR1 (as appropriate) and secondary HRP-conjugated antibody, prior to chemiluminsence detection. Images were developed using a Kodak OXmat M20 film processor onto Kodak Hyper ECL film.

### Purification of trisk95 and junctin from skeletal SR vesicles

Trisk95 and junctin were isolated by SDS preparative gel electrophoresis, as previously described [39].

### Ca^2+^ binding capacity

The Ca^2+^ binding capacities of CSQ1 constructs were determined using a modified ^45^Ca^2+^ spin dialysis binding assay [14]. In brief, 200 μg of CSQ1 constructs were conjugated with ^45^Ca^2+^ by incubation in 150 mM NaCl, 20 mM MOPS, 100 μM to 5 mM CaCl_2_ (pH 7.4), and 2.6 kBq ^45^Ca^2+^ for 15 min at room temperature. Unconjugated ^45^Ca^2+^ was removed by centrifugation of the samples in a Microcon centrifugal filter concentrator at 12,000 × *g* for 10 min. ^45^Ca^2+^ radioactivity of the retentate (containing CSQ1-^45^Ca^2+^) and of an unfiltered sample aliquot were counted using a Packard 1500 Tri-Carb liquid scintillation analyzer (Packard Instrument Co., Downers Grove, IL, USA). Protein concentration of the retentate (containing CSQ1-^45^Ca^2+^) was determined using a Bio-Rad DC protein determination assay, according to manufacturer’s instruction. Data is presented as nmol ^45^Ca^2+^/mg CSQ1.

### Co-immunoprecipitation

*In vitro* binding of CSQ1 to purified trisk95 and junctin was performed using a Pierce co-immunoprecipitation kit as per manufacturer’s instruction, with the following changes. Co-immunoprecipitation was performed in a buffer containing 20 mM MOPS, 150 mM NaCl, and 1 mM CaCl_2_ (pH 7.4), using a 2:1 protein ratio (weight) of CSQ1 to either junctin, trisk95, or RyR1.

### Affinity chromotography

CSQ1-RyR1 interactions were investigated using affinity chromatography as previously described [34].

### Turbidity measurements

Solution turbidity [40,41] was monitored spectrophotometrically in a 1-cm path length quartz cuvette. Three micromolar protein was suspended in a buffer containing 20 mM Tris and 100 mM KCl pH 7.4. Small aliquots of CaCl_2_ were added to the cuvette to final concentrations of 0.1 to 3 mM. After each addition, the cuvette was stirred and allowed to equilibrate at room temperature for 7.5 min, after which the absorbance was recorded at 350 nm. Absorbance values were corrected for any change in absorbance due to buffer alone. Turbidity is a measure of CSQ1 transition from soluble to insoluble forms as a function of [Ca^2+^], and increased absorbance at 350 nm reflects the shift toward insoluble particles.

### Single channels

Artificial planar bilayers separating two baths (*cis* and *trans*) were formed as described previously [7,34]. Native SR vesicles (50 μg) or purified RyR1 (10 μg) were added to the *cis* solution so that the cytoplasmic surface of the SR and RyR1 faced this solution after incorporation. Solution constituents were as follows: *cis* - 230 mM CsMS, 20 mM CsCl, 1 mM CaCl_2_, and 10 mM TES (pH 7.4) and *trans* - 30 mM CsMS, 20 mM CsCl, 1 mM CaCl_2_, and 10 mM TES (pH 7.4). Free [Ca^2+^] in all solutions was verified using a Ca^2+^ electrode. Single channel parameters were measured using the channel 2 program (developed by P.W. Gage and M. Smith, John Curtin School of Medical Research, Canberra, Australia). Single channel recordings were obtained at +40 and −40 mV, at 23 ± 2°C. Channel activity was assessed from 90 s of recording at each potential, by directly measuring open probability (*P*_*o*_), mean open time (*T*_*o*_), mean closed time (*T*_*c*_), and open frequency (*F*_*o*_) in single channel recordings using threshold discrimination or indirectly from the fractional mean current (*I*’_*F*_) when more than one channel was opening [9]. For simplicity, *P*_*o*_ and *I*’_*F*_ are combined in calculations of average *P*_*o*_ or relative *P*_*o*_ [9].

### Statistics

Average data are presented as mean ± SE. The significance of differences between control and test values was tested using a Student’s *t*-test for paired data. In some cases, to reduce the effects of variability in control parameters (*P*_*o*Con_, *T*_*oC*on_, *T*_*c*Con_, and *F*_*o*Con_) and to evaluate parameters after CSQ1 construct addition (*P*_*o*CSQ_, *T*_*o*CSQ_, *T*_*c*CSQ_, and *F*_*o*CSQ_), data were expressed as the difference between log_10_*X*_CSQ_ and log_10_*X*_Con_ for each channel (for example, log*P*_*o*CSQ_ − log*P*_*o*Con_). The difference from control was assessed with a paired *t*-test applied to log10*X*_Con_ and log10*X*_CSQ_. A *P* value of ≤0.05 was considered to be significant.

## Results

### Expression of CSQ1

CSQ1 contains a highly acidic-rich C-terminal domain, ^354^EDDDDEDDDDDDDD^367^, purported to be a Ca^2+^ binding motif, and contains residues essential for binding both junctin and trisk95 [15]. To investigate the function of the CSQ1 C-terminal domain, we generated two deletion mutants of GST-tagged rabbit CSQ1 and three alanine substitution mutants. The two CSQ1 deletion mutants were generated by deleting either the whole C-terminal tail (CSQ1_∆C14_) or 50% of the tail (CSQ1_∆C7_) (Figure 1A). As CSQ Ca^2+^ binding motifs consist of a pair of acidic residues located close in space (with each pair binding one calcium ion), alternate acidic residues were substituted to disrupt one (CSQ1_DA1_), two (CSQ1_DA2_), or three (CSQ1_DA3_) Ca^2+^ binding sites within the sequence (Figure 1A). For single channel and ^45^Ca^2+^ binding studies, CSQ1 constructs were cleaved from the GST by incubation with the serine endopeptidase Factor Xa and purified to homogeneity (Figure 1B).

### Effects of C-terminal tail modification on the Ca^2+^ binding capacity of CSQ

**Figure 2:**
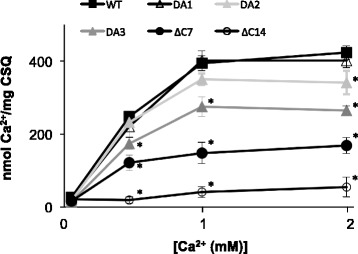
**CSQ1 Ca** ^**2+**^ **binding capacity.** The ^45^Ca^2+^ binding in a spin dialysis binding assay was carried out using 200 μg protein (at a concentration of 1 mg/ml) in 150 mM NaCl, 20 mM MOPS, 100 μM to 5 mM CaCl_2_, and 2.6 kBq ^45^Ca^2+^. Each data point is the mean ^45^Ca^2+^ bound, in nmol Ca^2+^/mg CSQ, and the bars are SEM for each of the CSQ1 constructs, under the Ca^2+^ conditions listed. Asterisks (*) indicate a significant difference in ^45^Ca^2+^ bound to CSQ1 constructs compared to WT CSQ1 (*P* < 0.05). *N* = 5 to 9.

The Ca^2+^ binding capacity of the CSQ1 constructs was determined using ^45^Ca^2+^ binding. Ca^2+^ binding capacity is dependent on CSQ1 structure and increases sharply as CSQ1 polymerizes. Given that CSQ1’s C-terminus is believed to form a large acidic Ca^2+^ binding pocket upon polymerization [27], it follows that disruption of this pocket would reduce Ca^2+^ binding capacity. Mutation of one or two acidic residues (CSQ1_DA1_ and CSQ1_DA2_) within the C-terminus had no significant effect on Ca^2+^ binding capacity from 0.1 to 1 mM Ca^2+^, and the binding curves were similar to that we have previously reported [42], but CSQ1_DA2_ bound significantly less Ca^2+^ than WT CSQ1 at 2 mM Ca^2+^ (Figure 2). Mutation of three acidic residues (CSQ1_DA3_) was sufficient to reduce Ca^2+^ binding capacity to approximately 70% of WT CSQ1 at resting [Ca^2+^] of 1 mM and to significantly depress binding capacity at all other [Ca^2+^] tested. Truncation of the C-terminus (CSQ1_∆C7_) led to a 40% to 60% drop in Ca^2+^ binding capacity at all [Ca^2+^] tested, while removing the entire C-terminus (CSQ1_∆C14_) all but abolished Ca^2+^ binding (Figure 2). This provides further strong evidence that many of the C-terminal acidic residues of CSQ1 are key to the formation of a Ca^2+^ binding pocket, which enhances CSQ’s Ca^2+^ binding capacity.

### CSQ1 aggregation

**Figure 3:**
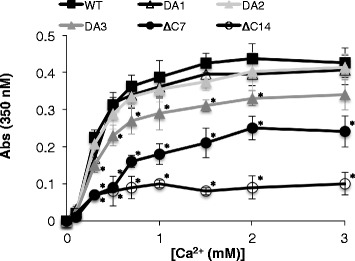
**CSQ C-terminal tail contributes to CSQ1 aggregation.** CSQ turbidity, an indicator of polymerization and aggregation, is measured as absorbance at 350 nm. Experiments were carried out in a buffer containing 20 mM MOPS, 100 mM KCl, and 0 to 3 mM CaCl_2_. Data is presented as mean ± SEM for each of the CSQ1 constructs. Asterisks (*) indicate average data significantly different (*P* ≤ 0.05) from that of WT CSQ1 at the same [Ca^2+^].

To determine whether mutation of the acidic C-terminus disrupted CSQ1 aggregation, turbidity measurements were performed. CSQ1 and CSQ2 undergo Ca^2+^-induced compaction or a shift from the proteins soluble to insoluble form [41]. CSQ1 aggregation is measured as turbidity (absorbance at 350 nm), which is proportional to the levels of insoluble CSQ1 [40,41]. The turbidity of WT CSQ1 increases sigmoidily across the [Ca^2+^] range tested, reaching a constant concentration of insoluble protein at between 1 and 1.5 mM Ca^2+^ (Figure 3). Mutation of one or two alternate residues yielded near identical results, while mutation of three residues resulted in a small approximately 20% decrease in absorbance across [Ca^2+^]’s of 0.7 to 2 mM (Figure 3; compare CSQ1_DA3_ with WT, CSQ1_DA1_ and CSQ1_DA2_). These data indicate that these residues do not play a major role in Ca^2+^-induced CSQ1 compaction. CSQ1_∆C7_ and CSQ1_∆C14_ displayed marked differences in the Ca^2+^-dependent aggregation of CSQ1 (Figure 3). CSQ1_∆C7_ showed some levels of Ca^2+^-induced aggregation; these were much less than WT, CSQ1_DA1_, CSQ1_DA2_, or CSQ1_DA3_, indicating that residues ^361^DDDDDDD^367^ are important in the formation of the higher molecular weight entities. Remarkably, deleting CSQ1 C-terminus severely disrupted CSQ1 ability to aggregate, presumably by disrupting Ca^2+^-induced polymerization.

### Effects of CSQ modification on *in vitro* interactions with trisk95 and junctin

**Figure 4:**
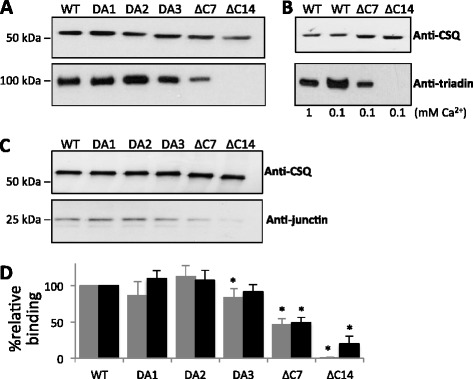
***In vitro*** **CSQ1 binding to trisk95 and junctin.** CSQ1 IP. Western blot showing binding of junctin and trisk95 to CSQ1 coupled to anti-CSQ1/protein A/G sepharose. **(A)** and **(C)**. Binding of trisk95 **(A)** and junctin **(C)** at 1 mM Ca^2+^ to the following CSQ constructs, from left to right; WT CSQ1; CSQ1_DA1_; CSQ1_DA2_; CSQ1_DA3_; CSQ1_ΔC7_, and CSQ1_ΔC14_. **(B)** Binding of WT CSQ1, CSQ1_ΔC7_ and CSQ1_ΔC14_ to trisk95 at 100 μM Ca^2+^. WT CSQ1 binding to trisk95 at 1 mM Ca^2+^ is shown in in the first lane for comparison. Each blot in **(A)** to **(C)** was immunoprobed with antibodies against CSQ1, trisk95, and junctin. Binding was repeated three to four times. **(D)** Quantitation of trisk95 and junctin association with CSQ1 C-terminal mutants at 1 mM Ca^2+^. The relative percentage binding of trisk95 (gray bin) and junctin (black bin) is presented as mean ± SE. Data is presented relative to the association of trisk95 and junctin with WT CSQ1. No band was detected for trisk95 binding to CSQ1_ΔC14_. Asterisks (*) indicate average data significantly different (*P* ≤ 0.05) from binding of trisk95 or junctin to WT CSQ1. Molecular weight markers are to the left of the blots in **(A)** and **(C)**.

We tested the ability of CSQ1 to associate with trisk95 and junctin via its C-terminal residues, using co-immunoprecipitation. Purified CSQ constructs were coupled to anti-CSQ1/agarose protein A/G, prior to exposure to trisk95 or junctin. As has been previously reported [42], there are significant interactions between WT CSQ1/trisk95 and WT CSQ1/junctin at the resting luminal [Ca^2+^] of 1 mM Ca^2+^ and ionic strength (150 mM) (Figure 4A,C first lane). A substantial interaction between the proteins remained after mutation of one or two alternate residues. There was a small but significant decrease in trisk95 association with CSQ1_DA3_ (Figure 4A,D), indicating that ^363^D may play a minor role in the trisk95-CSQ1 association. This result suggests that these three alternate C-terminal residues are not critical for the interaction with either trisk95 or junctin.

The deletion constructs CSQ1_∆C7_ and CSQ1_∆C14_ reveal that the overall C-terminal tail domain heavily influences trisk95’s interaction with CSQ1. Deletion of the entire C-terminal tail abolished CSQ1 association with trisk95 (Figure 4A,D). Interestingly, there is a significantly reduced band of trisk95 indicating minimal association with the CSQ1_∆C7_ construct (Figure 4A,D), indicating that residue ^361^DDDDDDD^367^ contributes significantly to the trisk95-CSQ1 interaction, with a minor contribution from ^363^D. The reduced CSQ1-trisk95 interaction may be due to altered Ca^2+^ sensitivity of the trisk95-CSQ1 interaction, which has been shown to be highly Ca^2+^-dependent [15]. There is only modest trisk95-CSQ1 binding at resting luminal [Ca^2+^] (1 mM) and much higher trisk95-CSQ1 association at lower Ca^2+^ concentrations (approximately 100 μM) [15]. However, lowering [Ca^2+^] to 100 μM did not significantly alter the degree of trisk95 association with CSQ1_∆C7_ or CSQ1_∆C14_ (compare CSQ1_∆C7_ and CSQ1_∆C14_ in Figure 4A (1 mM Ca^2+^) with CSQ1_∆C7_ and CSQ1_∆C14_ in Figure 4B (100 μM)). Therefore, the impaired interaction is not due to a shift in Ca^2+^ dependence of the association but most likely due to deletion of the interaction site on CSQ1 for trisk95.

In contrast to the effects on CSQ/trisk95 binding, deletion of the C-terminal tail of CSQ1 severely impeded, but did not abolish, junctin association with CSQ1 (Figure 4C,D). Both the CSQ1_∆C7_ and CSQ1_∆C14_ constructs bound approximately 50% to 80% less junctin than did WT CSQ1 (Figure 4C; compare WT with CSQ1_∆C7_ and CSQ1_∆C14_). This implies that the C-terminal tail forms a major part of the junctin binding motif but does not constitute the entire binding domain.

### Effects of CSQ1 modification on the regulation of RyR1 channels by CSQ1

The functional consequence of the CSQ1 C-terminal mutations on RyR1 regulation by CSQ1 was studied in lipid bilayers. At a physiological resting [Ca^2+^] of 1 mM, CSQ1 polymers are tethered to the RyR1 through its interactions with trisk95 and junctin, allowing CSQ1 to inhibit native rabbit skeletal RyR1, specifically through its association with junctin [39]. Thus, it is likely that mutation of residues that reduce Ca^2+^ binding capacity and/or inhibit interactions with junctin would alter CSQ1’s functional effect on RyR1 gating.

**Figure 5:**
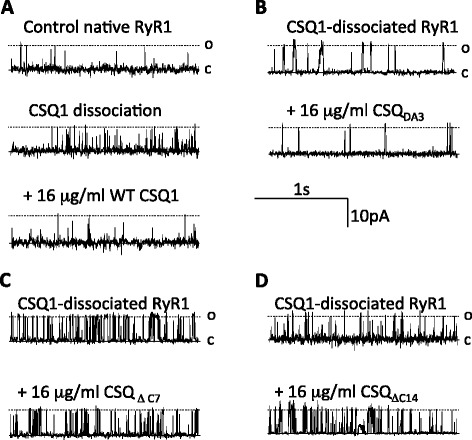
**Deletion of the C-terminal tail, but not sequential mutation, results in ablation of CSQ1 regulation of RyR1 gating. (A)** to **(D)**. Records of 3 s of single channel activity, where channel opening is upward from zero current (c, continuous line) to maximum open conductance (o, broken line) at +40 mV. **(A)** Top trace - control activity, with 2 mM *cis* ATP and 100 nM *cis* Ca^2+^ free. Middle trace - channel activity increased approximately 2 min after raising *trans* Cs to 500 mM, indicative of endogenous CSQ1 dissociation [33]. Bottom trace - following perfusion of the *trans* chamber with control *trans* solution, and subsequent addition of 16 mg/ml WT CSQ1. **(B)** to **(D**) Response of CSQ1-dissociated native RyR1 before (top) and after (bottom) addition of 16 mg/ml *trans* CSQ1_D∆3_ **(B)**, CSQ1_∆C7_ **(C)**, and CSQ1_∆C14_ **(D)**.

**Figure 6:**
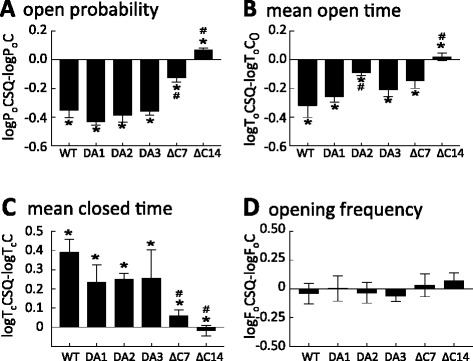
**Ablation of CSQ1-induced RyR1 regulation is due to significant changes in channel** ***P***_***o***_ **,** ***T***_***o***_ ***,*** **and** ***T***_***c***_ **.** Average relative data (relative to activity before the addition of CSQ1 constructs; *N* = 8) showing *P*_*o*_, *T*_*o*_, *T*_*c*_, and *F*_*o*_. **(A)** Relative *P*_*o*_ (log rel *P*_*o*_) is the average of differences between the log10 of *P*_*o*_ in the presence of CSQ1 constructs (log*P*_*o*CSQ_) and log10 of the control *P*_*o*_ (log*P*_*o*Con_; in the absence of CSQ1) for each channel. **(B)** The relative mean open time (log rel *T*_*o*_) is log*T*_*o*CSQ_ − log*T*_*oCon*_. **(C)** The relative mean closed time (log rel *T*_*c*_) is log *T*_*c*CSQ_ − log *T*_*cCon*_. **(D)** The relative mean open frequency (log rel *F*_*o*_) is log*F*_*o*CSQ_ − log*F*_*oCon*_. Average data significantly different (*P* ≤ 0.05) from channel activity recorded in the absence of WT CSQ1 is indicated by asterisk (*) and in the presence of WT CSQ1 indicated by crosshatch (#). Data were recorded at +40 and −40 mV.

Native skeletal rabbit SR vesicles (which contain the RyR1 and its full complement of associated co-proteins) were incorporated into lipid bilayers, and baseline activity was recorded after addition of 2 mM *cis* ATP and 4.5 mM *trans* BAPTA (to lower *trans* Ca^2+^ to 100 nM, that is, a sub Ca^2+^-activated level; Figure 5A, top panel). After approximately 3 to 5 min of stable baseline recording, the Cs^+^ concentration in the *trans* chamber was increased to 500 mM for 5 min to strip endogenous CSQ1 from the native RyR1 [7]. The *trans* chamber was then perfused with *trans* solution to remove dissociated CSQ1 and to reduce *trans* [Cs^+^] to 250 mM. Dissociation of endogenous inhibitor CSQ1 from RyR1 (to yield CSQ-dissociated RyR1) caused an increase in channel open probability (*P*_*o*_), which is sustained after the removal of high *trans* Cs^+^ (Figure 5A, middle trace). As previously reported [7], re-association of WT CSQ1 with the luminal face of the CSQ-dissociated RyR1, caused significant inhibition of RyR1 activity, restoring channel activity to the baseline level (Figures 5A and 6A).

Re-association of each of CSQ1_DA1_, CSQ1_DA2_, and CSQ1_DA3_ with CSQ-dissociated RyR1 also inhibited channel activity. Adding 16 μg/ml CSQ1_DA1_, CSQ1_DA2_, or CSQ1_DA3_ caused a 2.7-fold, 2.4-fold, and 2.2-fold decrease in channel activity, respectively (Figures 5B and 6A,B,C,D; Table 1). The degree of inhibition by these three constructs is reminiscent of the >2-fold decrease in channel activity induced by WT CSQ1 (Table 1 and [7]).

**Table 1:** **Comparison of changes in RyR1 channel gating parameters induced by association of CSQ1 constructs**

		**(** ***X*** **± SEM)**					
		**WT CSQ**	**CSQ1**_**DA1**_	**CSQ1**_**DA2**_	**CSQ1**_**DA3**_	**CSQ1**_**ΔC7**_	**CSQ1**_**ΔC14**_
RyR1-CSQ	*P*_*o*_	0.19 ± 0.02	0.19 ± 0.03	0.17 ± 0.07	0.15 ± 0.04	0.12 ± 0.01	0.12 ± 0.02
RyR1 + CSQ	*P*_*o*_	0.08 ± 0.01*	0.07 ± 0.01*	0.06 ± 0.02*	0.07 ± 0.01*	0.10 ± 0.01*#	0.15 ± 0.01*#
RyR1-CSQ	*T*_*o*_	4.93 ± 1.40	3.55 ± 0.26	3.44 ± 0.28	3.08 ± 0.42	3.80 ± 0.48	7.09 ± 1.99
RyR1 + CSQ	*T*_*o*_	2.43 ± 0.53*	1.96 ± 0.15*	2.78 ± 0.23*	1.99 ± 0.36*	2.67 ± 0.30*	6.70 ± 1.76#
RyR1-CSQ	*T*_*c*_	18.73 ± 4.02	25.03 ± 4.95	22.20 ± 6.40	33.85 ± 5.37	115.78 ± 38.29#	40.62 ± 7.81#
RyR1 + CSQ	*T*_*c*_	33.41 ± 4.97*	39.74 ± 10.72*	38.01 ± 5.78*	46.14 ± 5.86#*	115.69 ± 50.11#	36.95 ± 10.40
RyR1-CSQ	*F*_*o*_	34.15 ± 2.72	39.68 ± 3.27	41.78 ± 5.65	32.97 ± 5.95	16.9 ± 4.50#	26.18 ± 7.95
RyR1 + CSQ	*F*_*o*_	27.32 ± 5.66	36.37 ± 8.20	39.20 ± 7.20	27.76 ± 5.79	17.46 ± 4.40#	25.18 ± 6.49

Endogenous CSQ1 was first dissociated from RyR1. The first row for each parameter shows average data for channel activity following dissociation of endogenous CSQ1 (RyR1-CSQ1) and before addition of the recombinant CSQ1 construct. The second row in each parameter shows average data after addition of the indicated construct. Parameters are open probability (*P*_*o*_), open time (*T*_*o*_), closed time (*T*_*c*_), and open frequency (*F*_*o*_), which are the mean ± SE (*N* = 7 to 12) of combined data recorded at +40 and −40 mV. Asterisks (*) indicate average parameter significantly different (*P* ≤ 0.05) from that recorded in the absence of CSQ1, and crosshatch (#) indicates average parameter significantly different (*P* ≤ 0.05) from that recorded after addition of WT CSQ1.

The decrease in *P*_*o*_ in the presence of WT CSQ1 and alanine substituted mutants CSQ1_DA1_-CSQ1_DA3_ is due to a significant decrease in mean open time (*T*_*o*_) (Figure 6B; Table 1). Similar decreases in (*T*_*o*_) were observed upon reassociating the CSQ1 alanine mutants (Figure 6B; Table 1). There were also significant increases in mean closed times (*T*_*c*_) of the channels, in line with those recorded in the presence of WT CSQ1 (Figure 6C; Table 1). As a result of the opposing changes in *T*_*o*_ and *T*_*c*_, there was no change in the overall frequency of opening (Figure 6D).

In contrast to substitution within the final five C-terminal residues (CSQ1_DA3_), deletion of the final seven C-terminal acidic residues reduced CSQ1’s ability to inhibit RyR1 channels (Figures 5C and 6A,B,C,D; Table 1). While the CSQ1_∆C7_ mutant inhibited channels, the inhibition was significantly less than the potent inhibition caused by WT CSQ1 and CSQ1_DA1_-CSQ1_DA3_. The decrease in *P*_*o*_ was accompanied by significant changes in *T*_*o*_ and *T*_*c*_ (Figure 6B,C; Table 1). This data correlates with the reduced ability of this construct to induce Ca^2+^ compaction (Figure 3) and to bind to junctin (Figure 4), which communicates CSQ1 signaling to RyR1 [39]. Removal of the entire C-terminal tail abolished CSQ1’s ability to inhibit the RyR1 channels (Figures 5D and 6A,B,C,D; Table 1). Indeed, CSQ1_∆C14_ evoked a small but significant activation of RyR1. This activation is similar to the activation seen when CSQ1 is added to purified RyR1, that is, only seen when trisk95 and junctin are absent [7,43] and indicative of a direct, but probably non-physiological, interaction between CSQ1 and RyR1.

**Figure 7:**
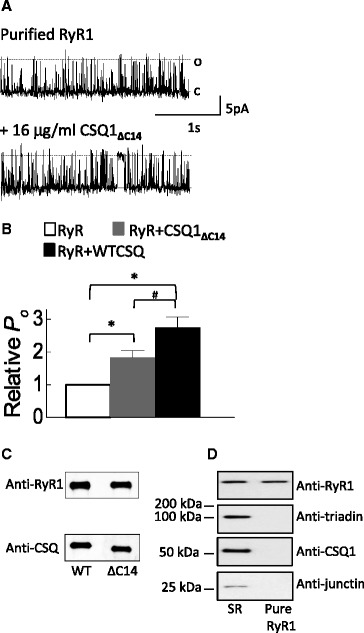
**CSQ1**_**∆C14**_ **binds to and activates purified RyR1. (A)** Records of 3 s of single channel activity, where channel opening is upward from zero current (c, continuous line) to maximum open conductance (o, broken line) at +40 mV. **(A)** Control purified RyR1 activity, with 2 mM *cis* ATP and 100 nM *cis* Ca^2+^ free (top trace) and after the addition of 16 μg/ml CSQ1_∆C14_. **(B)** Average relative data (relative to activity before the addition of WT CSQ1 or CSQ1_∆C14_; *N* = 9) showing open probability (*P*_*o*_). Average data significantly different (*P* ≤ 0.05) from channel activity recorded in the absence of CSQ1’s is indicated by asterisk (*). Crosshatch (#) indicates a significant difference (*P* ≤ 0.05) between the average relative *P*_*o*_ recorded in the presence of WT CSQ1 and CSQ1_∆C14_. **(C)** CSQ1 affinity chromotography. WT CSQ1 (left) and CSQ1_∆C14_ (right) after exposure to purified RyR1. Binding was repeated three times. Blot was immunoprobed with antibodies against RyR1 (top) and CSQ1 (bottom). **(D)**. Purification of RyR1 from SR vesicles. Immunoprobing purified RyR1 sample with anti-CSQ1, anti-trisk95, and anti-junctin shows no contaminant levels of these proteins in the purified sample. Molecular weight marker is between blots in **(C)** and **(D)**.

To investigate the nature of the small RyR1 activation by CSQ1_∆C14_, we examined its effect on purified RyR1 (Figure 7). There was no contaminant trisk95, junctin, or CSQ1 in this sample (Figure 7D), so that any effects of CSQ1_∆C14_ would be due to its association directly with RyR1, rather than via the anchoring proteins. Purified RyR1 activity rose significantly approximately 1.9-fold after the *trans* addition of 16 mg/ml CSQ1_∆C14_ (Figure 7A,B), although the degree of purified RyR1 activation was not as high as the approximately 2.8-fold activation induced by WT CSQ1 (Figure 7 and [7]). In addition, affinity chromatography shows that both the WT CSQ1 and CSQ1_∆C14_ bound to purified RyR1 (Figure 7C). This indicates that although there was no difference in the ability of CSQ1 to bind directly to RyR1 in the presence or absence of the C-terminal tail, full activation of RyR1 by CSQ1 depends on binding to residues in both the C-terminal domain of CSQ1 and in the N-terminal domains. We have previously suggested that despite CSQ1 causing a modest activation of purified RyR1 in the bilayer, it is the overwhelming inhibition of native RyR1 (through the CSQ1-junctin-RyR1 interaction; [39]) which drives the overall effect of CSQ1 in the cell, which would acts as a brake on SR Ca^2+^ release.

## Discussion

### Overview

Here, we present evidence that the C-terminal tail of CSQ1 forms the primary binding pocket for Ca^2+^ ions and that it contains the CSQ1 binding sites for trisk95 and junctin and for functional interactions with RyR1. This is also the first evaluation of the influence of specific acidic residues on the functional characteristics of CSQ1. Our novel data indicates the extent to which specific acidic residues in CSQ1’s C-terminal tail influence the Ca^2+^ binding capacity and Ca^2+^-induced aggregation of CSQ1, its ability to bind to trisk95 and junctin and its ability to inhibit RyR1. In summary, Ca^2+^ binding declines with progressive removal of acidic residues, and all 14 acidic residues are required for the full Ca^2+^ binding capacity of the wild-type protein. Deletion of one to three of the acidic residues has little effect on Ca^2+^-induced compaction or CSQ1 association with trisk95 and junctin and does not alter the inhibitory action on RyR1 of the CSQ1 binding to junctin. CSQ1 aggregation, binding of trisk95 and junctin to CSQ1, and the inhibitory effect of the binding on RyR1 activity are severely disrupted by deletion of seven of the acidic residues and are all but abolished by deletion of all 14 C-terminal residues.

### CSQ Ca^2+^ binding capacity

The Ca^2+^ binding capacity of CSQ1 decreased when two or more residues within the acidic C-terminal tail were modified or deleted. CSQ1’s moderate Ca^2+^ binding capacity has been thought to depend on CSQ1’s polymerization which occurs at relatively high [Ca^2+^] (>0.5 mM). This model is based on the published crystal structure of rabbit skeletal CSQ1 but is not supported by the crystal structure as residues 352 to 367 are not observed in the electron density mapping (reviewed in [33]). CSQ1 dimers and polymers condense at [Ca^2+^]s between 100 μM and 1 mM with a cellular ionic strength of 150 mM [6]. The Ca^2+^ dependence of CSQ1’s structure suggests that Ca^2+^ may act as a “glue”, interacting with key acidic residues when a polymer is formed. It is further hypothesized that residues within the C-terminus form a Ca^2+^ binding sink within the structured protein which is stabilized by the formation of three salt bridges (^215^E-^86^ K, ^216^E-^24^ K, and ^169^E-^85^ K) [27]. The linear sequence of full-length CSQ1 cannot account for its reported Ca^2+^ binding capacity, of 34 Ca^2+^ ions per monomer at resting (1 mM) [Ca^2+^] [4]. There are insufficient acidic residues on CSQ1 to form the 34 acidic doublets, assuming that one acidic double is required to bind each Ca^2+^ ion [44]. The Ca^2+^ sink hypothesis was recently refined, with simulation model data showing that Ca^2+^ preferentially saturates the C-terminal tail and induces protein polymerization which stabilizes the formation of many Ca^2+^ binding sites on CSQ1’s surface [32] which and could rationally account for the reported Ca^2+^ binding capacity. Our data provides the first experimental evidence supporting this refined hypothesis.

Furthermore, the mutation of three acidic residues in the C-terminal tail could only account for the loss of 3 of the 34 Ca^2+^ ions bound per monomer of CSQ1, yet Ca^2+^ binding data illustrates an approximately 45% loss of Ca^2+^ binding capacity. One interpretation of our data is that these residues are important in stabilization of the Ca^2+^ binding pocket formed upon CSQ1 polymerization in which the ratio of Ca^2+^ ions to acidic doublets residues is ≫ 1. This is supported by data showing CSQ1 structure and Ca^2+^ binding are drastically altered when the whole C-terminal tail is deleted (Figure 2 and [15,29]) and the inability of CSQ1_∆C14_ to form polymers (Figure 3) under our experimental conditions. CSQ1 is reported to be the key luminal Ca^2+^ sensor for the RyR1 and to guide RyR1’s response to the fluctuations in luminal [Ca^2+^] that occur during contraction and relaxation [6,8,34]. CSQ1 binds and releases Ca^2+^ in response to store load and to maintain free Ca^2+^ at approximately 1 mM and allows CSQ1 to sense the level of Ca^2+^ inside the SR. Given that mutation and deletion of the C-terminus has a profound effect on Ca^2+^ binding capacity, it is likely that the C-terminal tail is important in communicating luminal [Ca^2+^] to RyR1.

### Loss of CSQ1 binding to trisk95 and junctin

It was not surprising that the absence of the C-terminal tail impeded CSQ1-trisk95 association as the CSQ1 binding site for trisk95 has previously been localized to residues within the C-terminal tail [16]. Further dissection illustrates that residues 363, 365, and 367 do not play a pivotal role in this association, although comparison of binding of CSQ1_DA2_ and CSQ1_DA3_ do suggest a minor role for ^363^D. It appears that residues 354 to 362 are essential in forming CSQ1’s trisk95 binding site.

It is significant that a modest amount of junctin was able to bind to CSQ1_∆C14_, given that others have reported that the C-terminal domain of CSQ1 is the sole binding site for junctin in skeletal muscle [15]. This is consistent with the fact that more than one binding site on CSQ1 may be involved in its association with junctin in skeletal muscle, as with cardiac CSQ2 [13]. It is likely that a binding motif, in N-terminal domains of CSQ1, contributes to junctin association and would account the fraction of junctin binding remaining with CSQ1_∆C7_ and CSQ1_∆C14_.

### Loss of CSQ1 regulatory function of RyR1 channel activity

The significant decrease in CSQ1’s ability to inhibit RyR1 activity following deletion of 361 to 367, but that substitution of residues 363, 365, and 367 maintain normal RyR1 regulation implies that residues 361, 362, 364, and 366 contribute to maintaining the efficiency of CSQ1’s regulation of RyR1 [9]. The abolition of RyR1 inhibition by CSQ1 when the C-terminal tail was removed confirms both that this tail is essential in CSQ1’s role in inhibition of Ca^2+^ release through RyR1 and an important role for residues 354 to 360. CSQ1 inhibition of RyR1 activity is mediated via a RyR1/junctin/CSQ1 interaction, and not through RyR1/trisk95/CSQ1 [39]. Therefore, it is curious that CSQ1 inhibition of the channel is lost despite the remaining modest association of junctin with CSQ1_∆C14_. It is possible that CSQ1’s function as a channel inhibitor is directly related to its Ca^2+^ binding capacity and/or structure, which is severely reduced when the C-terminal tail is removed from CSQ1 (Figures 2 and 3).

### CSQ1 activation of RyR1 - not a physiological regulatory mechanism?

CSQ1_∆C14_ increases the activity of native RyR1 (Figure 6), and both WT CSQ1 [7,43] and CSQ1_∆C14_ activate purified RyR1 (Figure 7). It is likely that this activation is relatively unimportant physiologically, as full-length CSQ1 addition to RyR1 in the presence of anchors trisk95 and junctin results in a strong channel inhibition. In light of this, native RyR1 activation by CSQ1_∆C14_ can be explained in either of two ways. Firstly, WT CSQ1 associated with junctin exerts a dominant inhibitory effect on RyR1 gating, so that channel inhibition overshadows activation arising from the direct RyR1-CSQ1 association. When the binding site on CSQ1 for junctin is compromised in CSQ1_∆C14_, channel activation by the direct CSQ1-RyR1 coupling is unmasked. On the other hand, the binding site for WT CSQ1 on RyR1 may be occluded by CSQ1’s interaction with trisk95 and/or junctin. Thus, the reduced CSQ1_∆C14_ association with trisk95 and/or junctin may expose the binding site on RyR1 for CSQ1, allowing channel activation when CSQ1_∆C14_ is added to the *trans* chamber.

## Conclusions

CSQ1 is unarguably the major the Ca^2+^ buffer inside the SR. In addition, CSQ1 is both an inhibitor of native RyR1 under resting conditions and also a luminal Ca^2+^ sensor for the channel, so that it may act as a brake on RyR1 Ca^2+^ release in times of low store load [7,9]. As mentioned above, CSQ1 has been thought to regulate RyR1 via interactions with trisk95 and junctin. Our previous single channel data suggests that junctin, but not trisk95, is the key intermediate protein in RyR1 inhibition [39], although evidence from one trisk95 and junctin knockout study suggests that trisk95 is the preferential anchor for CSQ1 [45]. On the other hand, studies using myotubes deficient of trisk95 and junctin confirm our observations that junctin’s primary role is in communication of luminal Ca^2+^ to RyR1 [46]. It remains possible that anchoring and functional regulation are in fact separate processes. Regardless, CSQ1 undisputedly plays an important role in regulating RyR1 and in excitation-contraction coupling. The essential nature of CSQ1 is illustrated in CSQ1-null mice, which exhibit increased susceptibility to stress in a similar manner to exertional/environmental heat stroke and to human malignant hyperthermia (MH) a life-threatening hypermetabolic disorder induced by treatment with volatile anesthetics and the muscle relaxant succinylcholine [10,47,48]. This complex phenotype is likely initiated by abnormal Ca^2+^ handling by the SR, due to lack of CSQ1 inhibition on RyR1 activity [47].

## Abbreviations

Con: control
CSQ: calsequestrin
CSQ1: calsequestrin type 1
*F*_*o*_: open frequency
*I’*_*F*_: fractional mean current
kD: dissociation constant
*P*_*o*_: open probability
RyR: ryanodine receptor
RyR1: ryanodine receptor type 1
SR: sarcoplasmic reticulum
Trisk95: triadin skeletal muscle 95 kDa
*T*_*o*_: open time
*T*_*c*_: close time
